# Modulating the tumor immune microenvironment with nanoparticles: A sword for improving the efficiency of ovarian cancer immunotherapy

**DOI:** 10.3389/fimmu.2022.1057850

**Published:** 2022-12-01

**Authors:** Tianyue Xu, Zhihui Liu, Liwen Huang, Jing Jing, Xiaowei Liu

**Affiliations:** Laboratory of Integrative Medicine, Clinical Research Center for Breast, State Key Laboratory of Biotherapy, West China Hospital, Sichuan University, Chengdu, Sichuan, China

**Keywords:** immunotherapy, ovarian cancer, tumor immune microenvironment, nanoparticles, drug delivery system

## Abstract

With encouraging antitumor effects, immunotherapy represented by immune checkpoint blockade has developed into a mainstream cancer therapeutic modality. However, only a minority of ovarian cancer (OC) patients could benefit from immunotherapy. The main reason is that most OC harbor a suppressive tumor immune microenvironment (TIME). Emerging studies suggest that M2 tumor-associated macrophages (TAMs), T regulatory cells (Tregs), myeloid-derived suppressor cells (MDSCs), and cancer-associated fibroblasts (CAFs) are enriched in OC. Thus, reversing the suppressive TIME is considered an ideal candidate for improving the efficiency of immunotherapy. Nanoparticles encapsulating immunoregulatory agents can regulate immunocytes and improve the TIME to boost the antitumor immune response. In addition, some nanoparticle-mediated photodynamic and photothermal therapy can directly kill tumor cells and induce tumor immunogenic cell death to activate antigen-presenting cells and promote T cell infiltration. These advantages make nanoparticles promising candidates for modulating the TIME and improving OC immunotherapy. In this review, we analyzed the composition and function of the TIME in OC and summarized the current clinical progress of OC immunotherapy. Then, we expounded on the promising advances in nanomaterial-mediated immunotherapy for modulating the TIME in OC. Finally, we discussed the obstacles and challenges in the clinical translation of this novel combination treatment regimen. We believe this resourceful strategy will open the door to effective immunotherapy of OC and benefit numerous patients.

## Introduction

Ovarian cancer (OC) has a high lethality rate and is the second primary cause of death from gynecologic cancer worldwide (1). Currently, the major treatments for OC are surgery, chemotherapy, and radiotherapy (2, 3). Although patients can achieve short-term remission with these approaches, five-year survival rates are only approximately 30% (4). Recently, immunotherapy has received increasing attention, especially immune checkpoint blockade (ICB), which has emerged as an effective strategy for OC therapy. The anti-PD-1 antibody pembrolizumab has received regulatory approval to treat OC (5).

Although ICB holds tremendous potential for cancer therapy, the current clinical data on OC immunotherapy is not ideal. In general, the limited efficacy of ICB is mainly due to four reasons (1): tumor antigen deficiency (2), insufficient T lymphocyte infiltration, (3) defective tumor antigen processing and presentation mechanisms, and (4) the suppressive tumor immune microenvironment (TIME). Notably, the suppressive TIME is a significant barrier to the immunotherapy of OC. Ovarian tumors contain a large number of immunosuppressive cells, such as M2 tumor-associated macrophages (TAMs), CD4^+^ regulatory T cells (Tregs), myeloid-derived suppressor cells (MDSCs), and cancer-associated fibroblasts (CAFs), which inhibit the immune response.

In recent years, nanoparticles have been expected to play a significant role in regulating the TIME and improving the efficacy of OC immunotherapy. On the one hand, nanotechnology-mediated photothermal therapy (PTT) and photodynamic therapy (PDT) can induce immunogenic cell death (ICD) of tumor cells, promote antigen presentation, and enhance tumor T cell infiltration (6). For instance, copper sulfide nanoparticles remodeled the TIME by inducing ICD, thus improving the efficiency of immune checkpoint inhibitors (ICIs) in OC. On the other hand, nanoparticles can be used as excellent drug carriers, which can load immunomodulators, such as adjuvants, cytokines, and siRNA, to regulate immunosuppressive cells and inhibit immune checkpoints. For example, Kang Yanan et al. prepared liposomes containing toll-like receptor (TLR) agonists and successfully repolarized M2 TAMs in OC (7).

Above all, nanoparticle-mediated immunotherapy holds great promise in modulating the TIME of OC and improving the treatment outcome. Here, we summarized the composition and function of the TIME in OC and discussed recent advances in immunotherapy to treat OC in preclinical and clinical settings. Moreover, the advantages and progress of nanoparticle-mediated immunotherapy in regulating the TIME and boosting the antitumor immunity of OC are also summarized. Finally, the current limitations and future development strategies in clinical translation of this nanoparticle-mediated immunotherapy have also been discussed.

## The tumor immune microenvironment of ovarian cancer

### Overview of ovarian cancer

**Figure 1:**
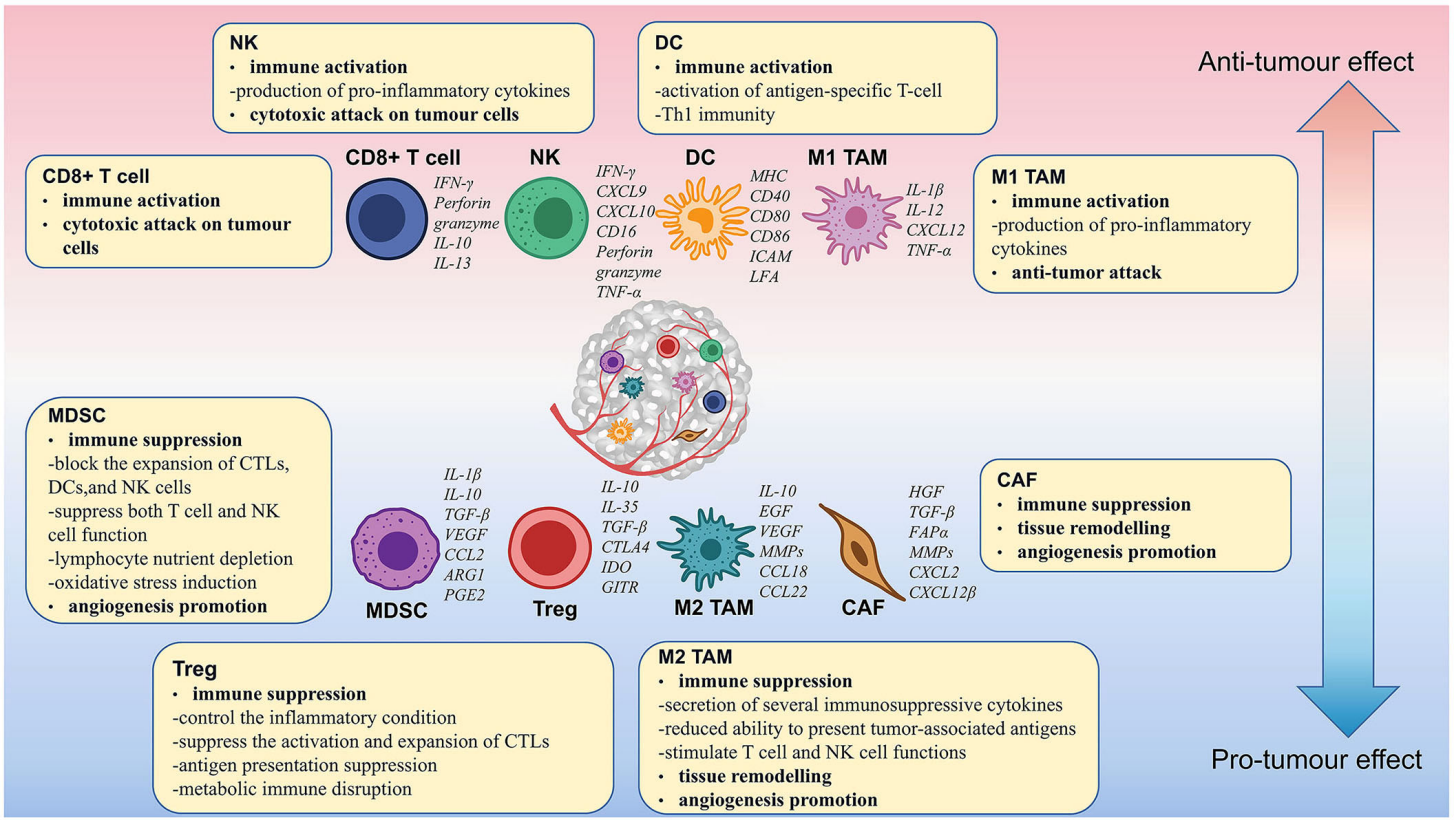
Immune cell functions and their interactions in the ovarian cancer tumor microenvironment. M2 TAMs, Tregs, MDSCs, and CAFs suppress the immune response and promote the proliferation, growth, and metastasis of OC. CD8^+^ T cells, NK cells, mature DCs, and M1 TAMs enhanced the immune response and suppressed tumor growth.

Approximately 90% of ovarian tumors originate in epithelial cells and are called epithelial ovarian cancer (EOC). EOC has been categorized into different subtypes according to histology. The prevalent histologic subtype is high-grade serous ovarian cancer (HGSOC), which accounts for about 80% of cases. Other rarer subtypes include low-grade serous, mucinous, clear cell, and endometrioid tumors. With the development of genomics and single-cell technology, the understanding of the TIME in OC has been deepened (8–12). It has been found that different OC subtypes have distinct macrophage polarization (13). The results of single-cell RNA sequencing revealed that ascites cells in different HGSOC patients differ in composition and functional program including diverse fibroblasts and macrophages (10). In addition, CD8^+^ and CD4^+^ T cells have distinct infiltration levels in differentially growing metastases within a single individual (9). Therefore, the TIME of OC is very complex and heterogeneous, which is primarily made up of CD8^+^ T cells, CD4^+^ T cells, NK cells, macrophages, MDSCs, etc. Based on their function, these cells can be categorized as activated and suppressive immune cells. Activated immune cells mainly include CD8^+^ T cells and NK cells. Suppressive immune cells mainly include Tregs, M2 macrophages, MDSCs, etc. In the TIME, activated immune cells play a role in tumor growth inhibition and tumor immunosurveillance. In contrast, suppressive immune cells dampen the function of activated immune cells and promote the growth of tumors (**Figure 1**).

### T lymphocytes

T lymphocytes are the main component of the TIME and are central to adaptive immunity. Mature T cells are classified as CD3^+^ CD8^+^ T cells and CD3^+^ CD4^+^ T cells, according to their marker gene (14). CD8^+^ T cells are the prime activated immune cells and are also known as cytotoxic T cells (CTLs). The T cell receptor (TCR) on CD8^+^ T cells binds to the MHC-I compound on tumor cells, resulting in the production of cytolytic factors (e.g., perforin and granzyme) and inflammatory cytokines (e.g., IL-2 and IL-12) that directly kill tumor cells (15). The mechanism of ICB and adoptive cell therapy (ACT) is to activate CD8^+^ T cells. An essential prerequisite for the PD-L1 blockade response in OC patients is sufficient T-cell infiltration. Higher infiltrating levels of CD8^+^ T cells in the TIME indicate a better prognosis in OC patients (16). However, high levels of TGFβ in OC inhibit the function of CTLs (17). Recently, it has been found that the infiltration level of CD8^+^ T cells in OC is regulated by CXCL9 expressed in antigen presentation cells (APCs) and CCL5 expressed in tumor cells (18).

In the suppressive TIME of OC, dysfunction of CD8^+^ T cells is another significant cause of immune dysfunction. T-cell dysfunction is caused by inhibiting T-cell mitochondrial biogenesis and the inability to produce sufficient energy intermediates (19). Activation of the IRE1α-XBP1 pathway regulates the T-cell mitochondrial activity and reduces T-cell infiltration and IFN-γ expression. Cytokine IFN-γ levels are linked to TIL infiltration, and increased IFN-γ levels can improve OC patient survival. Downregulation of XBP1 or control of endoplasmic reticulum stress enhances T-cell activity and metabolic adaptation (20). T-cell proliferation is also disturbed by lipid metabolites secreted by tumor cells, including 9-HODE, 5-HETE, and PGD2, which bind to T-cell PPAR and inhibit cell cycle protein E (21).

### Natural killer cells

NK cells are innate lymphoid-like cells with potent natural cytotoxicity against tumor cells. NK cells participate in immune regulation *via* a variety of mechanisms, including (1) the expression of CD16 to exert antibody-dependent cytotoxicity (ADCC) and detect target cells encapsulated in antibodies; (2) the production of perforin and granzyme to induce apoptosis in tumor cells directly; and (3) the release of antitumor cytokines such as TNF-α and IFN-γ (22). Various studies have demonstrated the effectiveness of NK cell therapy in patients with OC. Poznanski et al. found that expansion of patient-derived CD56^superbright^ CD16^+^ NK cells could exert potent cytotoxicity against autologous tumors in an autologous-derived xenograft mouse model of OC patients (23). Recently, Sun et al. showed that intravenous injection of NK cells isolated from the peripheral blood of OC patients inhibited the systemic metastasis of OC and increased the survival rate (24).

The most critical cytotoxic receptors for NK cells involved in immune surveillance are the NKG2D receptor, the CD16 receptor, and natural cytotoxic receptors for NKG receptors, such as NKp30 (25). In contrast, the proinflammatory cytokine MIF transcriptionally downregulates the NK cytotoxicity receptor NKG2D and decreases the cytotoxicity of NK cells (26). Furthermore, chronic receptor-ligand interactions reduce the expression of NK-cell surface receptors, impairing NK-cell cytolytic function and IFN-γ secretion ability (27). Greppi et al. found that OC cells released B7-H6 suppressed the expression of NKp30 on NK cells (28). TGF-β also inhibits NKp30 expression and dampens NK-cell-induced dendritic cell (DC) killing (27). In addition, OC ascites contain high levels of IL-18 and TGF-β, which can suppress the expression of CD16 and ADCC in NK cells (22). In OC, NK cells can also affect T cells to interfere with tumor progression. NK cells promote CD8^+^ T-cell recruitment in OC by upregulating CCL5, CXCL9, and CXCL10 *via* the CCR5 mechanism (29).

### T regulatory cells

Tregs are a heterogeneous subpopulation of CD4^+^ T cells that express CD25, CTL antigen 4 (CTLA-4), and the transcription factor FoxP3 (30). Tregs are classical immunosuppressive cells that exert immunosuppressive effects and maintain immune self-tolerance *in vivo*. Treg to CD8^+^ T cell ratios in tumors negatively correlate with survival in OC patients (31). Blocking Treg differentiation, migration, or immunosuppressive functions can reinforce the antitumor immune responses. Moreover, macrophage-secreted CCL22 in the peritoneal cavity can promote Treg migration (32). The hypoxic tumor microenvironment (TME) also favors the metabolic reprogramming of Tregs leading to Treg proliferation. Accumulated Tregs upregulate the secretion levels of IL-10 and promote angiogenesis and immune tolerance of tumors (33).

Tregs can establish a suppressive TIME through multiple mechanisms. On the one hand, Tregs can release immunosuppressive cytokines, such as IL-35, IL-10, and transforming growth factor β (TGF-β), which inhibit the function of CD8^+^ T cells and promote tumor cell growth (34). On the other hand, Tregs inhibit the TCR signaling pathway of CD4^+^ CD25- conventional T cells (Tcons), suppress calcium (Ca^2+^) signaling in Tcons, and reduce the activation of NFAT and NF-κB in Tcons (34). Moreover, the perforin and granzyme released from Tregs directly kill other immune cells, such as DCs, monocytes, and CD8^+^ T cells (34). Multiple receptors expressed on Tregs isolated from OC are associated with TCR involvement, including PD-1, ICOS, and 4-1BB. These receptors make Tregs more sensitive to anti-CD3/anti-CD28 stimuli and have a more potent inhibitory capacity (35). Treg-expressed CD73 and CD39 convert pro-inflammatory ATP to adenosine to mediate immunosuppression (36). CD4^+^ Tregs can differentiate into CD4^+^ effector T cells upon the activation of glucocorticoid-induced tumor necrosis factor receptor family-related receptor (GITR). Therefore, stimulation of GITR is expected to eliminate Treg-mediated suppression (37).

The secretion of high levels of proinflammatory cytokines (e.g., TNF and IL-6) in OC malignant ascites promoted high expression of tumor necrosis factor receptor 2 (TNFR2) in Tregs. TNFR2^+^ Tregs enhanced the expression of immunosuppressive molecules, including TGF-β, CD39, CD73, GARP PD-L1, and CTLA-4 (38, 39). The upregulated CTLA-4 in Tregs inhibits the activation and proliferation of effector T cells (40). Tregs in OC induce B7-H4 expression, deliver inhibitory activity to APCs, and blunt the antitumor immune response (41). Abnormal hyperactivation of signal transducer and activator of transcription-3 (STAT3) in tumor-infiltrating immune cells positively regulates the number of Tregs and MDSCs. Therefore, targeting the IL6/JAK/STAT3 signaling pathway is a feasible strategy to alleviate the immunosuppressive TME (42).

### Tumor-associated macrophages

TAMs are the largest immune cell population in the TIME of OC, accounting for 39% of the immune cellular repertoire. There are two main types of TAMs based on phenotype: proinflammatory M1-like and anti-inflammatory M2-like (43). M2 macrophages are associated with tumor immunosuppression in OC. In a study of 140 OC patients, Macciò and his colleagues found that a high density of M2 macrophages led to poor overall survival (OS) and prognosis. OS and the M1/M2 ratio were positively associated (13). Polarization and recruitment of M2 macrophages are key factors in OC progression and metastasis. The TIME can shift macrophages from the M1 to the M2 phenotype, creating a suppressive TIME. Ying et al. found that MiR-222-3p in the exosomes of EOC cells induces M2 phenotypic polarization through activation of the STAT3 pathway (44). Collagen triple helix repeat containing 1 (CTHRC1) is an OC secretory protein that induces M2-like polarization in TAMs by activating the STAT6 signaling pathway (45). Another reason for the poor survival and prognosis of OC is the recruitment of M2 macrophages. OC overexpressed UBR5, an E3 ligase, can recruit and activate TAMs by regulating multiple cytokines and chemokines, such as CCL2 and colony-stimulating factor 1 (CSF-1) (46). CSF-1 is a major macrophage survival factor. Targeting TAMs with anti-CSF-1R antibodies is a new therapeutic strategy for OC (47–49). Multiple cell signaling pathways can induce protumor and immunosuppressive properties in M2 TAMs, such as JNK, IL-13, IL-4, AMPK, PPARγ, IRF-3, IRF-4, and C/EBPβ (50).

The most common metastatic route for OC cells is the body cavity route, forming spheroids for metastasis. M2 macrophages are enriched in the omentum, which is the primary site of choice for OC metastasis. M2 macrophages secrete EGF to activate tumor cell EGFR and upregulate the VEGF/VEGFR signaling pathway to promote tumor cell proliferation and migration. In addition, EGF upregulates the expression of ICAM-1 and ɑMβ2 integrin in TAMs and facilitates the interaction between TAMs and tumor cells to form spheroids (51, 52). TAMs also secrete multiple cytokines and chemokines to reshape the suppressive TIME of OC and promote OC progression. El-Arabey et al. reported that TAMs promote the growth, migration, chemoresistance, and epithelial-mesenchymal transition (EMT) of TP53-mutated HGSOC cell lines by exosomes releasing GATA3 (43). Macrophage secretion of TNF-α induces MIF and EMMPRIN into tumor cells in an NF-κB- and JNK-dependent manner. Subsequently, macrophages release various MMPs to enhance tumor invasion, migration, and vascularization (53). TAMs also secrete IL-6 and IL-10, which activate the STAT3 pathway and promote tumor proliferation (54). TAMs secrete several chemokines, including CCL17, CCL22, and CCL18. These chemokines recruit Tregs and Th2 subsets and promote T-cell differentiation toward a Th2 phenotype (55).

### Myeloid-derived suppressor cells

MDSCs are a heterogeneous group of nonterminally differentiated myeloid cells with immunosuppressive properties. Consistent with other immunosuppressive cells, the infiltration of MDSCs is related to shorter OS in OC patients (56). High concentrations of several cytokines (e.g., IL-6, IL-10, IL-1β, VEGF, PGE2, and TNF-α) in the ascites of OC patients induce the accumulation of MDSCs (57). Growth factors G-CSF and GM-CSF promote the production of MDSCs by activating STAT3 and STAT5 signaling pathways and downregulating IRF-8 (58). Multiple chemokines (e. g, CCL1, CCL5, CCL7, CXCL8, and CXCL12) drive the recruitment of MDSCs to OC tumor sites *via* the CCR2, CXCR4, and CCR5 axes. Triggering of the CXCL12-CXCR4 pathway is controlled by the tumor-associated inflammatory mediator PGE2, and targeting PGE2 has the potential to block the migration of MDSCs into ascites (59).

Notably, MDSCs can increase the stem cell-like properties of OC cells. Li et al. found that induction of the CSF2/p-STAT3 signaling pathway by MDSCs could enhance the stemness of EOC cells (60). Cui et al. demonstrated that MDSCs induced microRNA101 expression and suppressed CtBP2, thereby enhancing the stem cell-like properties of OC (61). The immunosuppressive properties of MDSCs are dependent on PGE2-induced DNA methyltransferase 3 alpha (DNMT3A) upregulation and hypermethylation of myeloid genes (62). MDSCs have re-editable properties similar to those of macrophages. STAT3 inhibition and TLR signaling modulation can repolarize MDSCs and activate their immune function (63).

MDSCs generate a suppressive TIME by inhibiting the activities of activated immune cells. Previous studies found that MDSCs inhibit the activity and proliferation of NK cells and block the antigenic expression of DCs (56). In addition, MDSCs produce TGF-β, IDO, IL-10, and nitric oxide (NO) to reduce the proliferation and cytotoxicity of NK cells and exert immunosuppressive functions (64). The effects of MDSCs on NK cells were manifested by downregulating the expression of surface natural cytotoxicity receptors (NCRs), NKG2D, and DNAM-1 (65). Meanwhile, MDSCs can polarize M1 macrophages to the M2 phenotype and induce Treg amplification. MDSCs induce the activation and accumulation of M2 macrophages and stimulate the production of more IL-10. In turn, IL-10 can upregulate immunosuppressive factors, such as PD-L1 and Arg-1, inducing the activation and amplification of MDSCs (66). HIF-1 in the hypoxic environment of OC redifferentiates MDSCs into TAMs and promotes tumor progression (67). In addition, MDSCs secreted TGF-β and IL-10 can stimulate Treg migration and differentiation through CD40-CD40 L interactions (68).

MDSCs suppress the T-cell-mediated antitumor immune responses in the TIME of OC by multiple mechanisms (1): Depleting nutrients required by lymphocytes: MDSCs inhibit T-cell proliferation by upregulating ARG-1 to consume arginine and isolate 1-cysteine (69); (2) Restricting T-cell recruitment and inducing T-cell apoptosis by expressing Galectin9, AMPKα-1, and ADAM17 (70); (3) Regulating NO and ROS production and stimulate oxidative stress; (4) Production of peroxynitrite (PNT) inhibits the TCR signaling pathway: MDSCs produce peroxynitrite, which nitrates complexes in the TCR-CD8 complex when direct contact with T cells. Meanwhile, MDSCs disrupt the binding of CD8^+^ T cells to specific peptide-major histocompatibility complex (pMHC) dimers and inhibit T cell antigen recognition (71). (5) Secreting TGF-β enhances T-cell immunosuppressive phenotypic differentiation, such as promoting the differentiation of Th17, Th2, and Tregs (72). (6) Enhancing the expression of PD-L1 on tumor cells by the AKT/mTOR signaling pathway in a PGE2-dependent manner (73).

### Cancer-associated fibroblasts

CAFs are another major cell subpopulation in OC masses and play a crucial role in OC progression (74). Tumor cells are protected from immune surveillance by an extracellular layer of dense extracellular matrix (ECM) rich in fibronectin. However, this protective shell is produced by abnormal remodeling of the ECM and excessive deposition of fibroblasts. OC cells construct a strong protective barrier by reprogramming fibroblasts with miRNAs, mainly downregulating miR-214 and miR-31 and upregulating miR-155 (75). Overexpression of STAT4 in EOC cells depends on tumor-derived Wnt7a to induce the production of CAFs (76). NNMT regulates CAF differentiation, reduces histone methylation and s-adenosylmethionine, and supports OC proliferation, growth, and metastasis (77).

Fibroblasts are a key component of the basement membrane and peritoneum of the greater omentum in OC. CAFs recruit ascites tumor cells expressing high levels of alpha5-integrin (ITGA5) to form heterogeneous spheroids called MUs. Additionally, CAFs secrete EGF to maintain the MU structure by maintaining ITGA5 expression, which aids in the trans-somatic metastasis and OC peritoneal dissemination of HGSOC (78). Many markers activate CAFs, such as the extremely heterogeneous αSMA and FAP, and these markers help us develop new therapeutic targets for cancer (79, 80).

CAFs can remodel the ECM by secreting various cytokines as well as produce multiple paracrine signals with OC cells to induce OC cell growth, migration, and invasion. CAFs secrete DKK3 and activate YAP/TAZ and β-linked protein, the former inducing CAF tumorigenesis and the latter promoting OC invasion (81). CAF-derived POSTN promotes EMT by activating the PI3K/AKT pathway. It also promoted TGF-β1-induced activation of fibroblasts and invasion and migration of OC cells (82). TGF-β receptor type II and SMAD signaling upregulate VCAN and activate the NF-kB signaling pathway in CAFs. Alterations in these signaling pathways increase matrix metalloproteinase 9, CD44, and hyaluronic acid-mediated motor receptor expression in CAFs and promote the progression of advanced OC (83). The hepatocyte growth factor (HGF) is a key growth factor derived from CAFs. HGF stimulates OC cell growth and drug resistance by activating the c-Met/PI3K/Akt and GRP78 pathways (84). Fibroblast growth factor-1 (FGF-1) is another crucial factor in CAFs. FGF-1 regulates tumor progression by phosphorylating FGF-4, increasing the expression of Snail1 and MMP3, and activating the MAPK/ERK pathway (85). In addition, CAFs promote OC metastasis by secreting VEGF-A and tenascin-c (86).

Moreover, CAFs recruit immune cells and remodel the TIME *via* several cytokines and chemokines. Taki et al. found that CAFs produce the chemokines CXCL1 and CXCL2, which recruit MDSCs in OC (59). In addition, CXCL12β expression in CAF cells promotes the migration and differentiation of Tregs (87). CAFs interact with multiple immune components to regulate the immune activity of innate and adaptive cells and suppress antitumor immunity. Interleukin (IL)-1β is a major immunosuppressant in the TME and is significantly associated with CAF-expressed PS1. PS1 positively correlates with IL-1β levels under the regulation of the WNT/β-linked protein pathway. Inhibition of PS1 expression increases the proliferation and migration of CTLs and DCs (88). Browning et al. found that CAFs produce IL-6, a cytokine with protumorigenic function, which leads to severely poor prognosis and chemoresistance in OC patients (54).

## Current progress of immunotherapy in ovarian cancer

During the past years, various immunotherapies, including ICB, cancer vaccines, ACT, and cytokines, have been approved for OC treatment (**Table 1**). In this section, we will expatiate the progress of these treatments in OC.

**Table 1:** Clinical trials of immunotherapy for ovarian cancer.

	Interventions	Number	Phase	Status	Efficacy
Immune Checkpoint Blockade	Ipilimumab	NCT01611558	Phase 2	Completed	BOR: 15%
	Nivolumab	UMIN000005714	Phase 2	Completed	BOR: 15% ORR: 10% (1 mg/kg), 20% (3 mg/kg) PFS: 3.5 months OS: 20.0 months
	Pembrolizumab	NCT02674061	Phase 2	Completed	ORR: 7.4% (received 1-3 prior lines), 9.9% (received 4-6 prior lines) DCR: 37.2% (received 1-3 prior lines), 37.4% (received 4-6 prior lines)
	Pembrolizumab + Niraparib (PARP inhibitor)	NCT02657889	Phase 1/2	Completed	ORR: 18% DCR: 65%
	Durvalumab + Trabectedin	NCT03085225	Phase 1	Active, not recruiting	tumor shrinkage rate: 43% ORR: 21.4% 6-month PFR: 42.9%
	Pembrolizumab + Pegylated liposomal doxorubicin	NCT02865811	Phase 2	Active, not recruiting	CBR: 52.2% ORR: 26.1%
	Nivolumab + Bevacizumab (antiangiogenic agent)	NCT02873962	Phase 2	Recruiting	ORR: 40.0% (platinum-sensitive participants), 16.7% (platinum-resistant participants) PFS: 8.1 months
Cancer Vaccines	MUC1-targeted DC vaccine	NCT01068509	Phase 2	Completed	PFS:13 months (first clinical remission), >42 months (second clinical remission)
	Multivalent conjugate vaccine (MUC1-TN, GLO-H, GM2, TF) + OPT-821 (saponin-based immunoadjuvant)	NCT00857545	Phase 2	Completed	HR of PFS: 0.98 OS: 47 months
	Multiepitope FRα vaccine + durvalumab	NCT02764333	Phase 2	Completed	SD: 33.3% PR: 3.7%
	Oxidized whole-tumor lysate DC vaccine	NCT01132014	Early phase 1	Completed	SD: 52.0% PR: 8.0% 2-year OS: 100% (responders), 25% (nonresponders)
Adoptive cell therapy	TIL + lymphodepleting chemotherapy (cyclophosphamide and fludarabine) + IL-2	NCT02482090	Phase 1	Completed	3-month SD: 66.7% 5-month SD: 33.3% decrease in target lesions: 33.3%
	TIL + cyclophosphamide	/			CR: 14.3% PR: 57.1%
	TIL + lymphodepleting chemotherapy (cyclophosphamide and fludarabine) + IL-2 + Ipilimumab + Nivolumab	NCT03287674	Phase 1/2	Completed	12-month SD: 83.3% PR: 16.7%
	CAR-T targeting mesothelin	NCT02159716	Phase 1	Completed	BOR: 73.3%
Cytokines	Recombinant IL-2	/	Phase 2	Completed	ORR: 25.0%
	α-Recombinant interferon	/	Phase 3	Completed	CR: 36% PR: 9% PD: 55%
	IFN-γ + cisplatin + cyclophosphamide	/	Phase 3	Completed	CR: 68% 3-year PFR: 51% 3-year OS: 74%
	IL-2 + OK-432 + platinum- and Taxol-based chemotherapy	Case report	/	Completed	recurrence rate: 53.8% (immunochemotherapy), 88% (traditional chemotherapy)
	IL-18 + pegylated liposomal doxorubicin	NCT00659178	Phase 1	Completed	SD: 38% PR: 6%
	IL-2 + 13-cis-retinoic acid	/	Phase 2	Completed	5-year PFS: 29% OS: 38%

BOR, Best Overall Response Rate; ORR, Objective Response Rate; PFS, Progression-Free Survival; OS, Overall Survival; DCR, Disease Control Rate; PFR, Progression-free Rate; CBR, Clinical Benefit Rate; HR, Hazard Ratio; SD, Stable Disease; PR, Partial Response; CR, Complete Response.

### Immune checkpoint blockade

Effective immunotherapy relies on antigen presentation, inhibition of immunosuppressive cells, and activation of effector T cells. Among them, T-cell-mediated immune responses are crucial and modulated by inhibitory and stimulatory signals. Immune checkpoints regulate T cell activities and are closely related to tumor immunity. Currently, ICIs targeting CTLA-4 and programmed cell death protein 1 (PD-1) or PD-1 ligand (PD-L1) have achieved breakthrough results in clinical trials (89). Anti-PD-L1 antibodies (avelumab, durvalumab, and atezolizumab), anti-PD-1 antibodies (nivolumab and pembrolizumab), and anti-CTLA-4 antibodies (ipilimumab) have received FDA approval for the treatment of several malignancies represented by melanoma and non-small cell lung cancer (5, 90). However, the objective response rates (ORR) for single-agent ICIs in OC are only 6-15% (91). For example, in a phase II study of ipilimumab for patients with platinum-sensitive OC, the best overall response rate (BOR) was just 15% (NCT01611558). In addition, the BOR for platinum-resistant OC patients treated with nivolumab was 15%. In this clinical trial, 40% of the patients had grade 3 or 4 treatment-related side events (92). Besides, in a phase II clinical study of pembrolizumab in advanced recurrent OC (NCT02674061), the ORR was also less than 10% (93).

Due to the unsatisfactory efficacy of single-agent ICIs in OC, combination therapy has recently received much attention. Several studies have combined ICB with polyadenosine diphosphate ribose polymerase (PARP) inhibition, chemotherapy, and antiangiogenic therapy to improve the efficacy of OC immunotherapy (91). For example, pembrolizumab was combined with the PARP inhibitor niraparib for recurrent OC treatment. In this clinical trial, the ORR was 18%, and the illness control rate was 65% (94). Besides, the ORR for OC patients treated with durvalumab plus the anticancer drug trabectedin was 21.4% (95). In addition, patients with platinum-resistant OC responded well to pembrolizumab plus pegylated liposomal doxorubicin. In a clinical trial, 52.2% of the patients achieved a clinical benefit, and 26.1% experienced an overall response (96). Moreover, a phase II trial of nivolumab plus bevacizumab in recurrent OC patients was conducted. The results showed that platinum-sensitive patients had an ORR of 40.0% and platinum-resistant patients had an ORR of 16.7% (97). However, although combination therapy represents an approach to improving the efficacy of ICB, ICIs still demonstrated limited clinical activity for OC patients. The core reason is the suppressive TIME in OC, which leads to insufficient CTL activity. According to Grzywa TM et al., TAMs can decrease the amount of L-arginine in the TME and decrease T cell activation (98).

### Cancer vaccines

Cancer vaccines can promote antigen presentation by APCs and enhance the anti-tumor activities of antigen-specific CTLs. They have additional advantages in establishing immune memory and preventing tumor recurrence (99). At present, cancer vaccines, represented by DC vaccines, have achieved successful clinical results in the immunotherapy of various malignancies, including OC, melanoma, and prostatic cancer (100). DC vaccines that target MUC1 and NY-ESO-1 have been used to treat patients with OC. In a phase II clinical study of the MUC1-targeted DC vaccine for EOC patients, MUC1 T-cell-specific responses were observed but did not result in substantially increased progression-free survival (PFS) (101). Multiple antigens have been incorporated into cancer vaccines considering the negative effects of immune escape. However, this strategy still does not improve clinical outcomes for OC. For example, combining a multivalent conjugate vaccine (MUC1-TN, GLO-H, GM2, TF) with an adjuvant for patients with OC in the second or third clinical complete remission following chemotherapy did not prolong OS or PFS compared to adjuvant alone (102).

Taken together, although cancer vaccines can induce strong immune responses, their current clinical outcomes in OC have not been satisfactory. The main reason is the weak immunogenicity and suppressive TIME in OC. According to Schumacher et al., neoantigen recognition is uncertain in OCs, because of the inadequate mutational load and tumor heterogeneity (89, 103). At present, some strategies have been proposed to solve this dilemma. On the one hand, cancer vaccines can be combined with other treatment strategies, such as ICB. For example, patients with advanced platinum-resistant OC treated with the multiepitope FRα vaccine plus durvalumab achieved durable survival, with partial response rates of 3.7% and stable disease (SD) rates of 33.3% (104). On the other hand, incorporating as many tumor antigens as possible into cancer vaccines is a promising strategy to solve the heterogeneity of OC and improve treatment effectiveness (105). Tanyi JL et al. constructed an oxidized whole-tumor lysate DC vaccine for treating patients with platinum-treated recurrent OC. After administration, the vaccine stimulates T-cell responses and patients experience prolonged survival (106).

### Adoptive cell therapy

ACT is an immunotherapeutic regimen that harnesses autologous or allogeneic anticancer lymphocytes to promote tumor regression (105). ACT is mainly divided into three types: expanded natural TILs, chimeric antigen receptor T cells (CAR-T), and T-cell receptor engineered T cells (TCR-T) (107). ACT has achieved striking clinical success in various cancers, such as B-cell leukemias and melanoma. For example, patients with advanced melanoma responded favorably to ACT, with complete tumor shrinkage (108). However, despite several attempts, ACT has not achieved the desired effect for OC patients. Patients with recurrent or advanced EOC were treated with TILs following a single intravenous injection of cyclophosphamide. The results show that only 14.3% of the patients experienced a complete response, and 57.1% experienced a partial response (109). Besides, in a phase I clinical study of CAR-T targeting mesothelin (CAR-T-meso) for patients with OC, the CAR-T-meso cells showed limited clinical activity and short persistence (110).

The poor antitumor activity of ACT in OC is largely associated with the suppressive TIME. At present, several immunotherapies, such as ICB and cytokines, which can regulate the TIME, have been combined with ACT for OC to improve therapeutic activity. The combination of IL-2 with TILs was used to treat six patients with progressive platinum-resistant metastatic OC. There were 4 patients with SD for 3 months and 2 patients for 5 months (111). In another clinical trial, 83.3% of patients with late-stage metastatic HGSOC who received TILs, IL-2, ipilimumab, and nivolumab had SD for up to 12 months. The addition of ipilimumab improved T-cell proliferation positively impacted the T-cell phenotype and boosted CD8 T-cell tumor reactivity (112).

In conclusion, ACT has shown excellent potential in OC treatment, but its successful clinical application still faces obstacles. The physical barriers in OC limit the accessibility of CAR-T cells to tumor cells. Local CAR-T cell administration will offer solutions to this problem and improve antitumor efficiency (113). In addition, the small number of targeted antigens and their heterogeneous expression in ovarian tumors predispose them to antigen escape. Novel CAR-T cells simultaneously targeting multiple TAAs may increase the effectiveness of ACT in OC patients (114).

### Cytokines

A large class of tiny biomolecules known as cytokines plays a crucial role in cell signaling. Among them, IFNs, ILs, and chemokines have all been extensively utilized as immunomodulators to treat cancer (115). For instance, IFN-α has achieved FDA approval and is used to treat leukemia in clinical settings (115). In addition, IL-2 can cause complete and long-lasting tumor regression in patients with metastatic melanoma and renal cancer (116). Recently, cytokine-mediated immunotherapy has been evaluated in OC clinical studies but has not yet achieved excellent outcomes. A clinical study found that OC patients had a low response rate to a single intravenous injection of recombinant IL-12 (117). Besides, the overall response rate for platinum-resistant OC patients receiving intraperitoneal administration of IL-2 was 25% (118). Moreover, intraperitoneal injection of α-recombinant interferon (rIFN-α2) resulted in complete remission in 36% of patients with EOC but also induced significant toxic side effects (119). In addition, an adenoviral vector expressing IFN-β was used to treat two OC patients. One of the patients with distant metastasis and malignant pleural effusion achieved a complete response (120).

At present, cytokine-based immunotherapy has been combined with various antitumor therapies such as chemotherapy and antiangiogenic therapy to improve clinical efficacy. In OC patients, combining IFN-γ with first-line chemotherapy improved PFS while causing acceptable toxicity (121). IL-2 was combined with picibanil (OK-432) and traditional chemotherapy drugs for patients with advanced OC. These patients had a lower recurrence rate than patients receiving chemotherapy alone (122). In a clinical trial, patients with OC were treated with IL-18 plus pegylated doxorubicin liposomes. The results show that 6% of patients had a partial objective response, and 38% had an SD (123). In addition, the combination of 13-cis retinoic acid, which has antiangiogenic activity, with low-dose IL-2 was used to treat advanced OC patients. The patients had a 5-year PFS rate of 29% and an OS rate of 38%, with an increased number of lymphocytes and NK cells (124).

Above all, cytokines as excellent immunomodulators have shown exciting potential in combination therapy of OC. However, their low stability and short half-life essentially limit their application in the clinic. These issues are considered to be overcome by utilizing nanoparticle-based drug delivery systems.

## Nanomaterials for remodeling the immune microenvironment to enhance cancer immunotherapy

**Figure 2:**
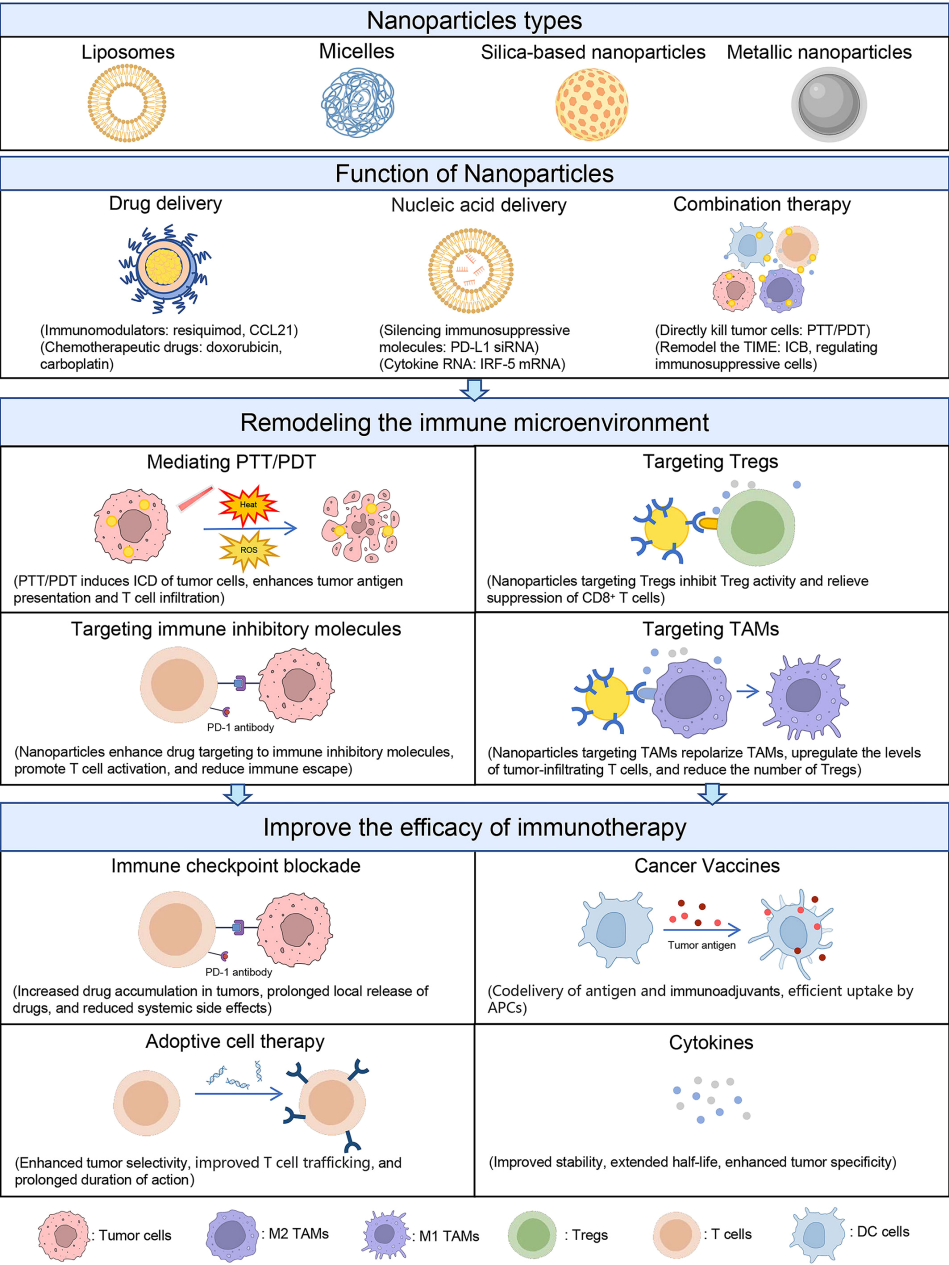
Schematic of nanoparticle-mediated immunotherapy regulating the TIME. Nanoparticles are mainly classified as liposomes, micelles, SNPs, and metallic nanoparticles. These nanoparticles have the functions of delivering drugs, delivering nucleic acids, and mediating combination therapy. Based on these functions, nanoparticles can regulate TIME in four ways: (1) mediating PTT and PDT to induce ICD in tumor cells; (2) improving drug targeting to immunosuppressive molecules; (3) targeting Tregs; and (4) targeting TAMs. By reversing the immunosuppressive state of TIME, nanoparticles can enhance the efficacy of immunotherapy such as ICB, ACT, tumor vaccines, and cytokines.

During the past decades, multiple nanoparticles have been applied in OC treatment or synergized immunotherapy, such as liposomes, polymeric micelles, silica-based nanoparticles (SNPs), and metal-based nanomaterials (**Table 2**). Nanoparticles not only serve as a vehicle to carry anticancer drugs but also as a regulator to modulate the TIME (**Figure 2**). In this section, we will discuss the role of nanoparticles in remodeling the TIME of OC and improving the efficacy of immunotherapy.

**Table 2:** Nanoparticles for regulating TIME and improving immunotherapy.

Nanoparticles	Immunotherapy	Targeting	Payload	Mechanism	Advantages	Ref
Liposome	PDT + ICB	PD-L1	IR775, metformin	PDT induces ICD; metformin downregulates PD-L1	Codelivery of hydrophilic and hydrophobic drugs	(125)
Liposome	Cytokines	TAMs	Resiquimod	TLR7/8 agonists repolarize TAMs	Administered intraperitoneally selective accumulation in TAMs	(7)
Liposome	ICB	Tregs	Indoximod prodrug, mitoxantrone	Indoximod inhibits the IDO-1 pathway and Treg expansion; mitoxantrone induces ICD	Codelivery of hydrophilic and hydrophobic drugs	(126)
Acid-sensitive polymeric nanoparticles	ICB + PDT	PD-L1	siPD-L1, carboplatin prodrug, digitoxin	Carboplatin prodrug initiates the caspase cascade; digitoxin elicits ICD; PD-L1 silencing overcome immune suppression	Environmentally responsive release and escape from the endocytic pathway	(127)
Biodegradable polymeric nanoparticles	Cytokines	TAMs	IRF5/IKKβ encoding mRNAs	IRF5 induces macrophage polarization; IKKβ activates IRF5	Reprogramming TAMs and safety for repeated dosing	(128)
PLG-g-mPEG nanoparticles	Cytokines	TAMs	Cisplatin, Resiquimod	TLR7/8 agonists repolarize TAMs	Passive targeting and drugs codelivery	(129)
Fusogenic lipid-coated MSNP	Repolarize TAMs	TAMs, PI3k	siRNA against PI3kγ, peptide LyP-1	Peptide LyP-1 targets TAMs; PI3kγ downregulation reprograms TAMs	Extremely high gene load and transfection efficiency, selective homing and transfection, avoidance of the endocytic pathway	(130)
Folic acid modified MSNP	Cytokines	T cells and DCs	CCL2	CCL2 recruits immune cells into the tumor tissue	Selective target-localizing ability and safety	(131)
SNPs	Repolarize TAMs	TAMs	/	Relatively large (>100 nm) anionic nanoparticles administered intraperitoneally selectively accumulate TAMs	Administered intraperitoneally selective accumulation in TAMs	(132)
Ferumoxytol capped ultra-large pore MSNP	ICB	PD-1	Anti-PD-1 antibody	Immune checkpoint inhibition	Sustained release and improved tumor specificity of ICIs	(133)
Copper chalcogenide nanoparticles	ICB + PTT	PD-1	Anti-PD-1 antibody, TLR9 agonist CpG	PTT induces ICD; TLR9 agonist CpG elicits activation of innate immune cells and adaptive immunity	Photothermal therapy with high penetration depth	(134)
Fe3O4 nanoparticles coated with a hybrid membrane consisting of ID8 ovarian cancer cell membrane and red blood cell membrane	PTT + PDT	/	Indocyanine green (ICG)	PTT induces ICD; red blood cell membrane coating improves the circulation time and stability; ID8 OC cell membrane coating support homologous homing properties	Prolonged circulation lifetime and high tumor specificity	(135)
Targeting peptide-modified gold nanoparticles	Inhibit TAMs	TAMs	siRNA against VEGF	siRNA inhibits the VEGF pathway in M2 TAMs and tumor cells, stimulating a host immune response	Selective gene silencing	(136)

### Application of liposomes in enhancing cancer immunotherapy

Liposomes are spherical bilayer nanoparticles composed of cholesterol and phospholipids, which have been used as drug vehicles due to their excellent encapsulation efficiency, targeting ability, biosafety, and biocompatibility (137). The remarkable properties of liposomes result in their FDA approval for use in clinical cancer treatment (138). In recent years, immunomodulators, such as adjuvants, photosensitizers, and tumor antigens, have been encapsulated in liposomes to regulate the TIME. For example, Xiong W et al. encapsulated the photosensitizer IR775 and metformin into liposomes. Under laser irradiation, the photosensitizer IR775 generates reactive oxygen species, which induce ICD in bladder and colon cancer cells and enhance antigen presentation (139). After PDT, the upregulation of IFN-γ amplifies the expression of PD-L1 on tumor cells (140, 141). The coencapsulation component metformin mediates the downregulation of PD-L1, which alleviates T cell exhaustion, synergistically enhancing the antitumor effect of PDT.

Since TAMs are the main immune cell population in OC, remodeling TAMs is a prospective strategy to improve the poor clinical outcomes of OC immunotherapy. TLR 7 and TLR 8 agonists, such as liquimod and resiquimod, serve as strong immunostimulatory molecules and have the ability to remodel TAMs (142). However, these small molecule drugs have serious toxicities when administered systemically. As an excellent target drug-delivery system, liposomes provide a way to deliver drugs into TAMs and remodel the TIME. For example, by loading resiquimod into liposomes, the drugs are efficiently delivered into TAMs and transformed M2 macrophages to the M1-type. Under treatment, the levels of tumor-infiltrating T cells were upregulated, while the percentage of Tregs in the TIME was reduced. When combined with PD-1 blockade, resiquimod-loaded liposomes significantly improve the antitumor efficiency of anti-PD-1 antibodies in OC (7).

Reducing the number of Tregs in the TIME is beneficial for promoting antigen presentation as well as T-cell recruitment and proliferation. Indoleamine 2,3-dioxygenase (IDO) is a metabolic immune regulator that can induce the expansion of Tregs (143). Kuo-Ching Mei et al. co-deliver cholesterol-conjugated indoximod prodrug, an inhibitor of the IDO-1 pathway, and chemotherapeutic agent mitoxantrone by liposomes into tumors. As a result, the number of Foxp3^+^ Tregs was obviously decreased, which in conjunction with chemotherapeutic drug-induced ICD, significantly boosted the immunotherapy response in multiple solid tumors (144). Therefore, liposomes encapsulating IDO pathway inhibitors can effectively reprogram the TIME by reducing the number of Tregs. This strategy is also expected to be successful in OC immunotherapy. Because a study has shown that IDO is widely expressed in 56% of ovarian tumors and is associated with decreased TIL numbers (125).

### Application of polymeric micelles in enhancing cancer immunotherapy

Polymeric micelles generally consist of a lipophilic core and a hydrophilic outer shell. Micelles have become widely used as drug carriers due to their biosafety, biocompatibility, surface modification, tumor targeting, and environmental responsiveness (145). These excellent biological properties have enabled micelles to be FDA-approved for the delivery of anticancer drugs (146). Recently, polymeric micelles have been used for drug delivery, bioimaging, and immunomodulation. Various immunomodulators, such as immunostimulants, immunoadjuvants, photosensitizers, and nucleic acids, have been entrapped into micelles to modulate the TIME (147). For example, FA-modified poly (ethylene glycol)-chitooligosaccharide lactate (COL) micelles were used as HIF-1a siRNA carriers. The micelles are efficiently taken up by cells *via* receptor-mediated endocytosis and significantly induce the transfection and gene knockout of HIF-1a *in vitro*, which effectively inhibits the proliferation of OC (148).

ICB can sensitize T cell-mediated tumor killing and has shown advantages in OC treatment. Genetic interventions, such as PD-L1 siRNA, are emerging as an effective strategy to suppress immune checkpoint signaling. However, the low transfection efficiency of gene therapy restrains its application as promising immunotherapy (126, 149). Cationic polymer micelles as excellent nucleic acid delivery vectors offer an attractive approach to boost genetic immunotherapy and improve the ICB response. Recently, Teo, P. Y. et al. loaded PD-L1 siRNA into folate (FA) or FA-polyethylene glycol (PEG)-modified PEI nanoparticles. The positively charged cationic polymer micelles facilitate the uptake of PD-L1 siRNA by interacting with negatively charged cell membranes. After administration, PD-L1 siRNA successfully transfected OC cells, effectively blocked PD-1/PD-L1 interaction, and enhanced the efficiency of ICB for OC (150). Recently, Ling, Xiang et al. constructed a pH-responsive nanocoordination polymer to deliver siPD-L1. This micelle was endocytosed into endocytic vesicles and ruptured when the endocytic vesicles transform into acidic endolysosomal, disrupting the organelle membrane and releasing siPD-L1 into the cytoplasm. Then PD-L1 was successfully knocked out for immune checkpoint inhibition, which remodeled the TIME and enhanced immune activation in OC (151).

Repolarizing M2 TAMs to the M1 phenotype is an effective strategy to remodel the TIME in OC and enhance antitumor immunity. Although various immunomodulators have been shown to repolarize TAMs, there are still many difficulties in repolarizing TAMs, such as poor targeting and the instability of immunomodulators. Due to the good surface modification, the polymeric micelles can be chemically bonded with diversified active targeting ligands to achieve specific targeting to TAMs. For example, mannose-modified polymeric micelles were used to deliver mRNA encoding IRF-5 and its activating kinase IKKβ. These polymeric micelles target mannose receptors overexpressed in M2 TAMs, delivering their payload exclusively to M2 TAMs. Following treatment, the immunosuppressive and tumor-promoting effects of M2 TAMs were successfully reversed (152). In addition, Yin Wen and his colleagues loaded the TLR7/8 agonist resiquimod and cisplatin onto poly(l-glutamate)-graft-methoxy polyethylene glycol (PLG-g-mPEG) nanoparticles. Benefiting from the protective function of these micelles, TLR agonists were successfully delivered and induced repolarization of macrophages, resulting in a synergistic anticancer effect of chemotherapy and macrophages in OC (153).

### Application of silica-based nanoparticles in enhancing cancer immunotherapy

SNPs are one of the most important nanomaterials applied in biomedical applications because of their excellent biocompatibility, biosafety, easy synthesis, and surface modification (154, 155). SNPs are mainly divided into three types: spheres, core-shells, and mesoporous SNPs (MSNPs) (156). Typically, MSNPs have many empty pores and large surface areas, which endow them as good candidates for drug delivery, bioimaging, and immune regulation. Immunomodulators, such as adjuvants, photosensitizers, cytokines, and siRNA, can be loaded into SNPs to regulate the TIME. For example, LyP-1 peptide-modified SNP-loaded siRNA against PI3K-γ can target TAMs and significantly knock down PI3K-γ expression (the knockdown efficiency is 81%), which leads to TAM polarization and remodels the TIME of OC (157). In addition, the surface modifiability of SNPs endows them can be wrapped with polymers and tumor-targeted peptides. These modified SNPs can enhance the drug delivery ability and avoid the toxicity of anticancer drugs. In our previous study, we developed tumor cell-targeted MSNPs by conjugating the indicated PAA and PEG on their surface (154). The MSNPs can selectively deliver MEK inhibitors into tumor cells instead of T-cells. MSNP encapsulation avoids the cytotoxicity of MEK inhibition on T cells and improves the antitumor efficiency of anti-PD-1 antibodies. The results suggest that MSNPs can avoid small molecule drug-induced immune toxicity and coordinate tumor-targeted therapy and immunotherapy.

Cytokines are classical immunoregulators that have been applied to treat OC. However, their rapid biodegradation and short half-life limit their clinical application. Uniform and large pore diameters as well as the easy surface modification ability endow MSNPs with high loading capacity, making them a candidate vehicle for carrier cytokines. In addition, the enhanced permeability and retention (EPR) effect of nanoparticles causes the carried cytokines to accumulate in TME and enhances their antitumor efficiency. Wimalachandra DC et al. developed an FA-modified SNP to load CCL21. Upon injection, CCL21-loaded SNPs accumulated at the TME of OC, which further recruited immune cells into the tumor tissue (127). Haber et al. found that negatively charged SNPs with a particle size larger than 100 nm administered intraperitoneally selectively accumulated in TAMs in mouse ovarian tumors (128). These results demonstrated that SNPs could serve as a candidate drug delivery system to remodel TAMs and enhance the anti-OC immune response by loading immune regulation agents.

ICB has been demonstrated to be an effective immunotherapy strategy and approved for the clinical treatment of OC. However, systemic toxicity and low local concentrations still need to be addressed. MSNPs have extremely high drug loading and can achieve controlled drug release by surface modification, making them ideal candidates for the delivery of ICIs. Bongseo Choi and his colleagues loaded an anti-PD-1 antibody into the pores of MSNPs and blocked the pores with iron oxide ferumoxytol, finally realizing the sustained release of PD-1 at the tumor site. This MSNP-mediated local ICB treatment after chemotherapy effectively promotes T cell infiltration and reduces Treg numbers in the TIME. This result indicates that MSNPs can achieve sustained release of ICIs and improve the duration of action and tumor specificity of ICIs (129).

### Application of metallic nanoparticles in enhancing cancer immunotherapy

Metallic nanoparticles are a kind of novel nanomaterial composed of pure metals (e.g., gold, silver, copper, iron, platinum, etc.) or their compounds (e.g., hydroxides, oxides, sulfides, etc.) (158). In recent years, metallic nanoparticles have been widely used for bioimaging and cancer treatment because of their excellent optical polarizability, electrical conductivity, biocompatibility, chemical properties, and potent photothermal properties induced by near-infrared (NIR) lasers (159). Metallic nanoparticles can mediate tumor PTT and PDT. PTT/PDT is an effective strategy to remodel the TIME and improve the efficiency of immunotherapy (160, 161). The mechanism of metallic nanoparticle-mediated PTT is that metal elements absorb light energy and convert it into heat to destroy malignant cells. Moreover, metallic nanoparticle-mediated PTT and PDT induce ICD in tumor cells, releasing tumor antigens and damage-associated molecular patterns (DAMPs) to stimulate the tumor-specific immune response and enhance immunotherapy (130). For example, gold nanoparticle-mediated PTT has been widely used alone or in combination with immunotherapy, chemotherapy, and targeted therapy to treat malignant tumors. In our previous study, we constructed a MAPK pathway inhibitor-loaded silica-modified gold nanocage (AuNCs) for synergistic melanoma therapy with an anti-PD-1 antibody. AuNC-mediated PTT along with MAPK-targeted therapy effectively kills tumor cells and enhances T-cell infiltration. This treatment regimen significantly improved the antitumor efficiency of PD-1 immunotherapy in the immune “cold” tumor and abscopal tumor models (131).

Although metallic nanoparticles mediated PTT/PDT has achieved gratifying antitumor efficiency in shallow tumors (e.g., melanoma), it has not reached the desired therapeutic effect in OC. In an OC mouse model, PTT alone did not inhibit tumor growth or prolong survival (132). The reason is that (1) the complex suppressive TIME and (2) the OC tumors located in a deep part of the human body prevent a laser from irradiating the tumor. Recently, Qizhen Cao and his colleagues developed copper monosulfide (CuS) nanoparticles to mediate a pulsed wave (PW) laser that can treat OC. CuS nanoparticles mediate photothermolysis, resulting in tumor cell death and improving the antitumor efficiency of PD-1 immunotherapy by promoting antigen presentation and T-cell infiltration (133). In addition, Xiong J et al. developed Fe_3_O_4_ nanoparticles coated with hybrid biomimetic membranes, which were formed by the fusion of red blood cell membranes and mouse-derived ID8 OC cell membranes. These metallic nanoparticles can extend the circulation half-life as well as homologous target ID8 OC cells and show synergistic PTT. Under this treatment, the tumor-specific antigens were released, which further improved the efficiency of immunotherapy by activating CD8^+^ CTLs and decreasing Foxp3^+^ Tregs (162).

Since the TIME in OC is also an important reason for limiting the effectiveness of PTT, combining strategies to modulate the TIME is a promising strategy to improve the effectiveness of PTT. The surface modifiability of metallic nanoparticles allows them to be coated with polymers and serve as vehicles for immunomodulators, such as adjuvants, cytokines, and siRNA, to regulate the TIME. For example, João Conde et al. constructed targeting peptide-modified gold nanoparticles encapsulated with siRNA against vascular endothelial growth factor (VEGF). Gold nanoparticles can selectively silence VEGF expression in tumor cells and TAMs, inhibiting immunosuppressive M2 TAMs (163). This strategy to remodel the TIME is expected to improve the antitumor effectiveness of PTT in OC.

### Application of other nanoparticles in enhancing cancer immunotherapy

Besides the nanoparticles introduced above, many other nanoparticles, such as carbon-based nanomaterials (CNMs) and metal-organic frameworks (MOFs), have also been reported to enhance cancer immunotherapy. CNMs, including carbon nanotubes, carbon dots, and carbon nanohorns, have gained attention in biological applications (164). Among them, carbon nanotubes have been explored as photothermal transduction agents and drug delivery carriers due to their surface modification, enhanced cellular internalization, electronic and optical properties, and biocompatibility (165). Carbon nanotubes can mediate PTT and induce ICD in tumor cells, and serve as delivery vehicles for tumor antigens and immunoadjuvants (166). For example, Yong Li et al. constructed annexin A5- modified single-walled carbon nanotubes for synergistic metastatic breast cancer therapy with an anti-CTLA-4 antibody. The nanoparticle-mediated PTT enhanced the abscopal response of ICB and increased the 100-day survival of tumor-bearing mice (167).

MOFs are novel nanoparticles composed of metal ions or clusters and organic ligands (134). The properties of MOFs, such as high porosity, large surface areas, surface modification, and luminescence characteristics, endow them as good candidates for drug delivery and diagnosis agents (135). Recently, various immunomodulators, including immunoadjuvants, tumor antigens, photothermal agents, and sonosensitizers, have been encapsulated in MOFs to modulate the TIME and synergize with immunotherapy (136, 161, 168, 169). For instance, Jiali Luo et al. developed cancer cell membrane-coated triphenylphosphonium decorated MOFs. The MOF-loaded sonosensitizer facilitated antigen presentation by mediating sonodynamic therapy. Co-delivered TLR agonist R387 promoted DC maturation. When combined with ICB, this nanoplatform finally reversed the suppressive TIME and enhanced the antitumor efficacy of immunotherapy (168).

## Conclusion and prospects

With a more in-depth understanding of the TIME, immunotherapy, especially ICB, tumor vaccines, ACT, and cytokines, has gained extensive attention in the treatment of OC. Although these immunotherapies have achieved excellent results in a variety of tumors, they are not ideal for the treatment of OC. This is mainly due to the suppressive TIME in OC, including Tregs, TAMs, MDSCs, and CAFs, which inhibit the antitumor immune response. Therefore, using smart strategies to transform the suppressive TIME into an antitumor state is of great significance for increasing the effectiveness of OC immunotherapy and extending patient survival.

In recent years, with the development of nanomedicine, immunotherapy combined with nanomaterials to modulate immune stimulation has achieved excellent preclinical and clinical efficacy. Mainstream nanoparticles include liposomes, micelles, SNPs, and metallic nanoparticles. Based on the excellent properties of nanoparticles in biocompatibility, drug loading, targeting capability, surface modification, and photothermal conversion, they have been widely used for regulating immune response and immunotherapy. On the one hand, nanoparticles can deliver immunomodulators that regulate the TIME of OC. On the other hand, photosensitizer-loaded nanoparticles or metallic nanoparticles can mediate PDT/PTT to induce the ICD of tumor cells, promoting antigen presentation and T-cell infiltration. These advantages make nanoparticles promising candidates for modulating the TIME and improving OC immunotherapy.

However, the toxicity, specific tumor targeting, and effectiveness of nanoparticles also need to be considered in clinical translation. In terms of toxicity, nanoparticles can not only interact with organism’s cells or blood cells but also produce toxic ions by the dissolution of nanomaterials. Through these mechanisms, nanoparticles exert toxicity leading to the damage of cells or vital enzymatic functions (170). Several strategies hold promise for reducing these toxic effects of nanoparticles. The nanoparticle can carrier negative surface charges and attenuate nanoparticle-cell interaction through modification with ligands, such as PEG (171). Covering the shell material or minimizing the surface area can reduce the dissolution of toxic ions (170). For specific tumor targeting, the poor manifestation of the EPR effect in the clinic and the nanoparticle-protein complex formed in systemic circulation cause off-target effects. Specifically, the differentials between animal models and human tumors and the complex TEM contribute to the failure of EPR-mediated targeting delivery in clinical translation. Several strategies, including enhancement of vascular permeability and depletion of tumor extracellular matrix, provide a chance to minimize the gaps between theoretical expectation and clinical outcome (172). Otherwise, serum proteins and opsonins are easily adsorbed on nanoparticle surfaces, which is probable to mask targeting ligands. Based on a deeper understanding of the interactions between nanoparticles and organisms, consideration of the protein corona effect when designing nanoparticles could improve the targeting efficiency (173). In terms of effectiveness, inefficient and unstable drug loading results in low drug concentrations in the TME and insufficient therapy. The limited light penetration depth also restricts the anti-tumor effects of photosensitive nanoparticle -mediated PTT. To improve the antitumor efficiency, nanoparticles with high pore volume and novel loading strategies have been developed that are beneficial for the enrichment of drugs at tumor sites (174). Some other approaches, including improving the photothermal conversion efficiency and developing NIR-II window PTT, are promising strategies to enhance the efficiency of PTT (175).

In this review, we discussed the impact of the TIME in OC on immunotherapy and mentioned the role of nanoparticles in modulating the TIME and improving the immunotherapeutic efficacy of OC. Using multiple approaches to overcome current shortcomings, we expect to leverage nanoparticle-based drug delivery systems to provide opportunities for the clinical application of OC immunotherapy.

## Author contributions

All authors listed have made a substantial, direct, and intellectual contribution to the work and approved it for publication.

## Funding

This work was funded by the National Natural Science Foundation of China (No. 22105137 and No. 82172634), Key Program of the Science and Technology Bureau of Sichuan (No. 2021YFSY0007), China Postdoctoral Science Foundation (No. 2020M683324 and No. 2022T150449), 1.3.5 project for disciplines of excellence, West China Hospital, Sichuan University (No. ZYYC20013).

## Conflict of interest

The authors declare that the research was conducted in the absence of any commercial or financial relationships that could be construed as a potential conflict of interest.

## Publisher’s note

All claims expressed in this article are solely those of the authors and do not necessarily represent those of their affiliated organizations, or those of the publisher, the editors and the reviewers. Any product that may be evaluated in this article, or claim that may be made by its manufacturer, is not guaranteed or endorsed by the publisher.
